# Methadone Prescribing for Pain Management in Pennsylvania per the Prescription Drug Monitoring Program, 2016–2020

**DOI:** 10.7759/cureus.28583

**Published:** 2022-08-30

**Authors:** Jenna R Adalbert, Karan Varshney, Jeffrey Hom, Asif M Ilyas

**Affiliations:** 1 Department of Orthopedic Surgery, Sidney Kimmel Medical College, Thomas Jefferson University, Philadelphia, USA; 2 Department of Public Health, Jefferson College of Population Health, Philadelphia, USA; 3 Division of Substance Use Prevention and Harm Reduction, Philadelphia Department of Public Health, Philadelphia, USA; 4 Rothman Orthopedic Institute Foundation for Opioid Research and Education, Thomas Jefferson University, Philadelphia, USA

**Keywords:** opioid use, opioid medication, pain management, prescription drug monitoring program (pdmp), methadone

## Abstract

Introduction

Methadone is a schedule II opioid traditionally used to treat opioid use disorder (OUD) and chronic pain. However, following the identification of its contribution to opioid overdose deaths, methadone has become less commonly used for chronic pain indications. In Pennsylvania (PA), prescribers are required to report methadone prescriptions written for pain indications to the prescription drug monitoring program (PDMP), which is an electronic database that enhances the tracking and reporting of prescription data. The primary objective of our study was to describe the geographic methadone prescribing trends recorded by the PA PDMP in order to report methadone’s current use for only pain indications.

Methods

State- and county-level methadone prescription data summaries recorded by the PA PDMP for each calendar quarter from August 2016 through March 2020 were collected from the PA Department of Health. The metric reported per quarter consisted of the total number of methadone prescriptions dispensed for pain indications unrelated to OUD.

Results

A total of 341,949 methadone prescriptions were dispensed in PA from the third quarter (Q3) of 2016 to the first quarter (Q1) of 2020 (range = 1106) with an overall 38.7% decrease in methadone prescriptions and a change in the rate of 85.97 per 100,000 population. The counties with the five highest prescription totals were Philadelphia, Allegheny, Bucks, Montgomery, and York (range = 46,969), and the counties with the five highest rates per 100,000 were Montour, Green, Columbia, Northumberland, and Forest (range = 964).

Conclusions

Methadone prescribing for pain management unrelated to OUD has decreased in PA from 2016 to 2020 per the PA PDMP. However, it is still prescribed in appreciable amounts for pain management. Further studies are required to understand the prescribing rationale and potential areas for harm reduction interventions.

## Introduction

The United States (U.S.) opioid epidemic is a public health crisis, with opioid overdose ranking prominently as a leading cause of injury-related death in the U.S. [1]. At the core of the epidemic exists the prescription opioid contribution, with provider fear of undermanaging patient pain and an absence of opioid prescribing guidelines perpetuating opioid morbidity and mortality [2,3]. To combat crisis severity, prescriber-level interventions have included studies proposing specialty- and procedure-specific prescribing guidelines [4] and opioid management recommendations from regulating bodies [5]. Subsequently, assessment of opioid intervention effectiveness at decreasing prescribing and regulation of prescribing practices has been promoted through the legislative development of state-specific prescription drug monitoring programs (PDMPs). While PDMP requirements for prescriber consultation and dispensary reporting are heterogenous between states, the general purpose of these statewide databases is to track controlled substance prescribing [6].

For opioid medications specifically, PDMPs offer harm reduction benefits by identifying risky provider prescribing behaviors and directing provider attention to patients at an increased risk of opioid overdose due to concomitant prescriptions [7]. In particular, methadone is a potent schedule II opioid medication, traditionally recognized as the standard for maintenance treatment of opioid use disorder (OUD) [8]. Methadone can also be used for pain management in those without OUD following its authorization in the 1990s. However, after the ensuing popularity as an opioid analgesic, methadone became a considerable contributor to prescription drug overdoses, primarily attributed to its complex pharmacologic properties and variable efficacy in achieving pain relief among patients [9]. Once aware of these risks, regulating bodies issued multiple warnings to prescribers and the public. In 2006, the Food and Drug Administration issued an official “black box” warning following a sharp rise in overdose deaths. In the following three years, the US Drug Enforcement Administration and the Government Accountability Office released reports highlighting mounting deaths from methadone urging, methadone makers to limit sales to hospitals and addiction clinics [10]. As a result, in 2013 North Carolina became the first state to remove methadone from its preferred drug list, followed by 16 other states taking similar action in the following years [10]. However, despite ongoing caution from regulating bodies and subsequent regulatory changes from other states, methadone has remained a preferred opioid medication for chronic pain in Pennsylvania (PA) state and insurance formularies due to its cost-effectiveness [10]. While records of methadone prescriptions supplied for OUD are protected by confidentiality regulations [11], prescriptions indicated only for pain require mandatory provider reporting to the majority of state PDMPs, including the PA PDMP.

Accordingly, the purpose of this study was to analyze and describe the prescribing trends of methadone for pain management unrelated to OUD in Pennsylvania (PA) from August 2016 to March 2020, as reported by the PA PDMP. Given the unique safety considerations that accompany methadone prescribing, we aim to evaluate and publicly report these trends in the context of methadone risks and benefits for pain management to increase patient and provider awareness.

## Materials and methods

This retrospective, cross-sectional study using de-identified, aggregate patient data were determined exempt from human subject review by the Thomas Jefferson University Institutional Review Board. 

Data source

The PA PDMP is an electronic, statewide database monitored by the PA Department of Health (DOH) and established to collect information on schedule II-V substance prescriptions filled at a dispensary [11]. For opioids specifically, the PA DOH utilizes the statewide PDMP to collect and record data on several opioid prescribing metrics at the state- and county-level. Prior to October 2014, the PA PDMP was operated by the Office of the Attorney General, with mandatory reporting limited to schedule II substances [11]. Following Act 191 of 2014 [12], operations and development of the statewide PDMP were transferred by the PA legislature to the PA DOH, which launched a new PDMP system in August 2016. Beginning January 1, 2017, the state government mandated that all licensed dispensers and practitioners in PA report each dispensed schedule II-V prescription to the PA PDMP [13]. In addition, all licensed prescribers with authorization to distribute, dispense, or administer controlled substances in PA were required to register for the PDMP and consult the database at select times to support safer prescribing decisions [13].

Data metrics and analysis

For the purposes of this study, the PA DOH provided de-identified state- and county-level methadone prescription data summaries reported by the PDMP for each calendar quarter from August 2016 through March 2020. The metric reported per quarter consisted of the total number of methadone prescriptions dispensed for pain indications unrelated to OUD. Geographic information system (GIS) mapping was used to depict county-level data on total prescriptions and prescription rates [14] with the use of ARcGIS version 10.8 (ESRI, 2020). Prescription rates were calculated using state- and county-level population data from the U.S. Census Bureau [15]. Data were extracted, tabled, and graphed for a longitudinal review of methadone prescribing trends for the overall state of PA and each individual county.

## Results

Table 1 details the number of methadone prescriptions dispensed and reported for each calendar quarter (August 2016 to March 2020) by the PA PDMP.

**Table 1:** Methadone prescriptions were reported for each calendar quarter (August 2016 to March 2020) by the PA PDMP. PA: Pennsylvania, PDMP: prescription drug monitoring program.

Quarter and year	Total prescriptions	Prescription rate (per 100,000 people) (as per 2019 data)
Q3: 2016	28,414	221.95
Q4: 2016	27,222	212.64
Q1: 2017	26,445	206.57
Q2: 2017	25,967	202.84
Q3: 2017	24,808	193.78
Q4: 2017	24,142	188.58
Q1: 2018	22,983	179.53
Q2: 2018	23,258	181.67
Q3: 2018	22,299	174.18
Q4: 2018	21,449	167.54
Q1: 2019	19,869	155.20
Q2: 2019	19,689	153.80
Q3: 2019	19,175	149.78
Q4: 2019	18,366	143.46
Q1: 2020	17,408	135.98
Total	341,494	2667.51

Total prescriptions

**Figure 1:**
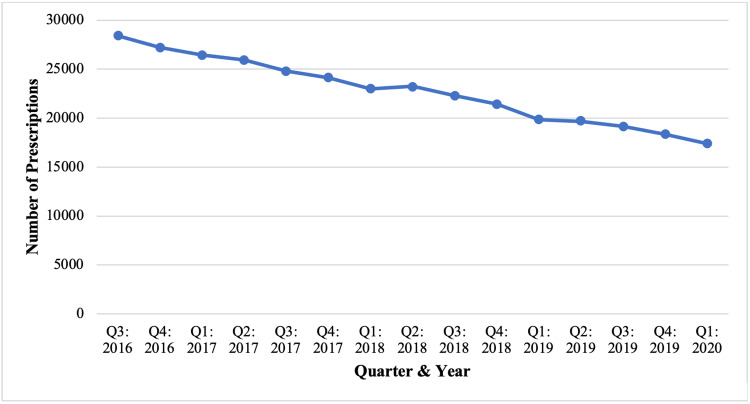
Total number of methadone prescriptions dispensed in PA from August 2016 to March 2020 per the PDMP. PA: Pennsylvania, PDMP: prescription drug monitoring program.

Overall, a total of 341,949 methadone prescriptions were dispensed in PA from the third quarter (Q3) of 2016 to the first quarter (Q1) of 2020 (Table 1). Prescriptions ranged from 28,414 in Q3 of 2016 to 17,408 in Q1 of 2020, reflecting a relatively consistent decline in prescribing (Figure 1). During this interval, a 38.7% decrease in methadone prescriptions dispensed for pain indications was observed in PA per the PDMP.

Rate of prescriptions

**Figure 2:**
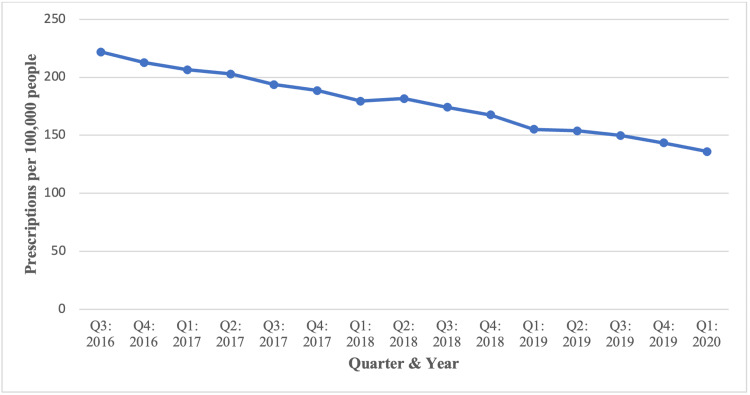
Rate of methadone prescriptions (per 100,000 people) across PA from August 2016 to March 2020 per the PDMP. PA: Pennsylvania, PDMP: prescription drug monitoring program.

The rate of methadone prescriptions (per 100,000 people) is depicted in Figure 2 and listed in Table 1. Prescription rates demonstrated a continual decline throughout the observed period. The prescription rate was highest in Q3 of 2016, at 221.95, and lowest in Q1 of 2020, at 135.98. The average prescription rate from Q3 2016 to Q1 2020 was 177.8. 

Methadone prescriptions by county

**Figure 3:**
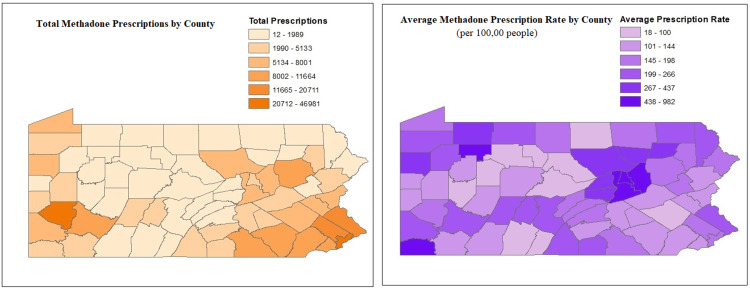
Dispensed methadone prescription totals and average rate per 100,000 population for PA counties from August 2016 to March 2020 per the PDMP. PA: Pennsylvania, PDMP: prescription drug monitoring program. Authors' own creations.

**Figure 4:**
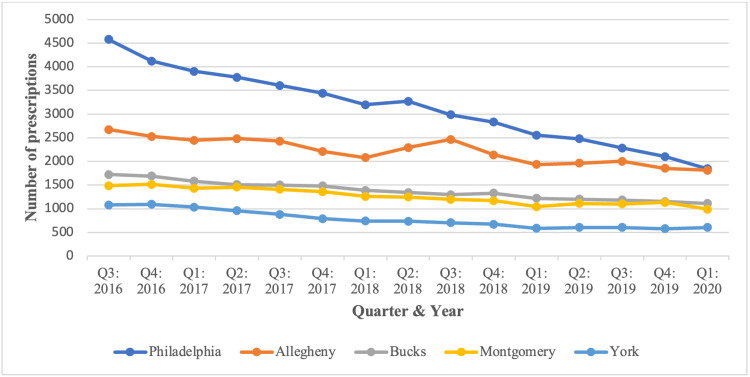
Total number of methadone prescriptions from August 2016 to March 2020 for the five highest counties in PA. PA: Pennsylvania.

**Figure 5:**
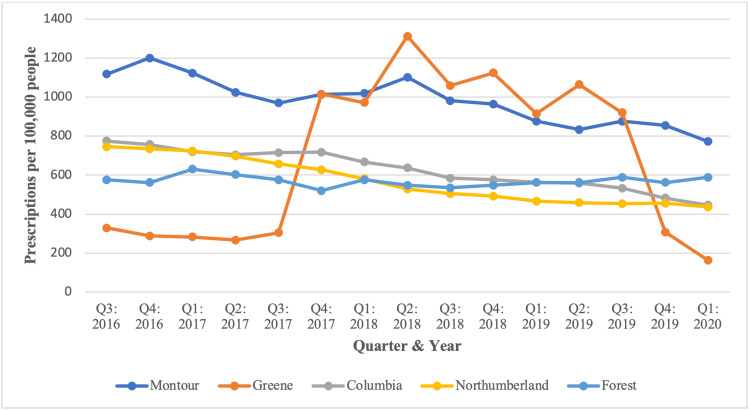
Rate of methadone prescriptions (per 100,000 people) across PA from August 2016 to March 2020 for the five highest counties. PA: Pennsylvania.

Figure 3 depicts dispensed methadone prescription totals and rates for all PA counties. Prescription totals ranged from 12 to 46,981, and the average prescription rate (per 100,000 people) ranged from 18 to 982 dispensed methadone prescriptions. The counties with the five highest prescription totals were Philadelphia, followed by Allegheny, Bucks, Montgomery, and York. Despite recording the highest totals, none of these five counties had the highest average prescription rates. Instead, the counties with the five highest rates were Montour, followed by Green, Columbia, Northumberland, and Forest County. Totals for these counties are listed in Table 2, and a breakdown by quarter for each of these counties is listed in Figures 4, 5.

**Table 2:** Counties with the highest methadone prescription averages by total and rate (per 100,000 people) in PA from August 2016 to March 2020. PA: Pennsylvania.

County	Mean prescriptions	County	Mean rate (per 100,000)
Philadelphia	3132.07	Montour	973.45
Allegheny	2221.73	Greene	629.11
Bucks	1380.73	Columbia	628.28
Montgomery	1260.93	Northumberland	570.86
York	777.60	Forest	552.31

## Discussion

Our longitudinal review of the PA PDMP data reveals a notable decrease in methadone prescribing for pain indications only and unrelated to OUD from 2016 to 2020. In accordance with U.S. Drug Enforcement Agency (DEA) data, our findings support decreasing methadone prescribing trends for pain indications observed in both PA and nationally [16,17]. Of note, regional disparities in decreasing trends exist, with the Northeast (including PA) decreasing 7.77% less than the Midwest, 11.84% less than the South, and 12.02% less than the West from 2017 to 2019 [17]. Yet overall, following the recognition of overdose potential and lethality of methadone, PA and national methadone prescribing trends for pain management indications have decreased progressively since 2006 [17,18]. 

Despite this decrease, the number of methadone prescriptions written annually in PA for indications other than OUD warrants consideration. While methadone accounted for approximately 1% of all opioid medications in the U.S. prescribed for pain in 2014, it comprised more than 23% of prescription opioid overdose deaths that year [19]. This disproportionate contribution to prescription opioid mortality is related to two specific characteristics of methadone: cost and drug properties [20]. Methadone’s appeal exists within its cheaper, generic selections that offer financial advantages for healthcare payers, as well as its potency, safety in renal insufficiency, and long-acting properties that decrease daily dosing requirements for patients with chronic pain conditions [9,19]. However, these pharmacologic properties are also responsible for methadone’s lethality and comprise the rationale for expert supervision during drug administration. While methadone can provide pain relief for up to eight hours, its analgesic effects are often delayed following administration, and its highly variable elimination half-life in patients can range anywhere from 30 to 60 hours [19]. As a result, patients may prematurely administer extra doses to achieve pain relief or accumulate excessive amounts of methadone in their system once the analgesic effects have subsided [21]. Additionally, the interpatient elimination inconsistencies result in difficulties with safely calculating methadone titration, and there remains no universal method for converting patients from other opioids to methadone [22]. Excessive methadone administration can quickly result in toxic levels of the drug, causing lethal respiratory depression and cardiotoxicity [9].

Recognition of methadone’s mortality risks and the minimal evidence for benefit in patient analgesia [23] has emphasized its inappropriate status as the first-line drug of choice for chronic pain management, influencing several states to remove methadone from their preferred drug lists [10]. However, its role as maintenance therapy for OUD remains paramount in reducing the morbidity of the opioid epidemic. Adherence to methadone treatment results in significantly reduced mortality rates for individuals with OUD [24], and the setting of administration for medication-assisted therapy (MAT) differs importantly from prescriptions for pain management. Methadone is administered as MAT to opioid-tolerant patients at treatment programs under the direct supervision of an expert provider [25], while the majority of providers prescribing for pain management are primary care or mid-level providers (i.e., nurse practitioners) that also treat opioid-naïve patients [26], increasing the risk of overdose. While a federal confidentiality law (CFR part 2, subpart C) exists in the U.S. to protect addiction treatment records [22] and prevents providers from reporting methadone prescriptions for OUD to the PDMP, the Substance Abuse and Mental Health Services Administration has recently opposed this lack of reporting [27]. Altering this federal legislation introduces negative legal and stigma-related implications for individuals seeking addiction treatment that requires careful consideration. However, transparent methadone reporting may improve our analyses of methadone indications contributing to overdose deaths and our understanding of related public health metrics. For example, as safer MAT alternatives (such as buprenorphine) emerge as effective treatment options for individuals with OUD [28], methadone reporting to the PDMP for OUD may inform our ability to select treatments for specific populations based on the success of patient management trends.

While the direct success of PDMPs at reducing opioid prescribing and overdose deaths remains widely debated [29], their potential as a reliable database for tracking prescribing patterns may be a key opioid harm reduction measure. At present, significant diversity exists between state PDMPs, with all 50 states exhibiting remarkable diversity between reporting, provider consult, and data-sharing requirements [30]. We emphasize that the PA PDMP’s mandatory reporting structure enabled our longitudinal review of methadone prescribing trends for pain management, and relays important implications for future PDMP data analysis. Given the severity of our opioid crisis, the collaborative action of states implementing mandatory reporting for controlled substances creates a twofold advantage: (1) transparent and consistent data reporting for trend analysis and future harm reduction studies, and (2) a nationally linked database available for provider consult to safely manage patients that travel between states. 

Limitations

To our knowledge, this is the first study to directly report PA PDMP data on methadone prescribing trends. While the PDMP data enables us to report gross methadone prescribing trends for pain indications in PA, we cannot further explore contributing variables such as physician specialty, prescribing rationale, or types of patient pain and caution any assumption that this prescribing is inappropriate. However, the number of methadone prescriptions identified for pain indications in our PDMP review period is a key consideration for future public health interventions related to optimal pain management strategies and overdose deaths. This study also does not compare the PA prescribing trends to that of surrounding states. This would aid in illustrating regional trends to possibly identify causes for discrepancies, and perhaps clue in on national trends. Additionally, the PDMP data may be a valuable tool for continuing to monitor methadone and other opioids commonly implicated in overdose deaths to inform potential areas of intervention.

## Conclusions

After performing a longitudinal review of dispensed methadone prescriptions reported to the PA PDMP database from 2016 to 2020, we conclude that methadone prescribing for pain management has decreased in PA. Despite minimal benefit in the context of its risks, pain management remains a significant indication for methadone prescriptions in PA. Future studies are required to further understand the provider rationale informing national methadone prescribing trends and identify important areas for methadone harm reduction interventions given that it is still prescribed in appreciable amounts for pain management.
